# Supplementary material for: OCTAVA: An open-source toolbox for quantitative analysis of optical coherence tomography angiography images
Source: PLoS One. 2021 Dec 9;16(12):e0261052. doi: 10.1371/journal.pone.0261052 (PMC8659314; doi:10.1371/journal.pone.0261052)
Supplement: S1 File — (DOCX) [file pone.0261052.s001.docx]

# S1 File. Optimization of Frangi “vesselness” filter

We investigated the parameters of the *Frangi “vesselness”* *filter* to optimize the visibility and characterization of vessels within the image [40,59]. The Frangi filter selectively enhances the intensity of individual pixels based on their “vesselness,” which is quantified using the eigenvalues of a local Hessian matrix $H\left( x,y,\sigma\right)= \sigma^{2}I\left( x,y \right)\frac{\partial^{2}}{\partial x\partial y}G(x,y,\sigma)$, where $I(x,y)$ is the intensity of a pixel in the image and $G\left( x,y,\sigma\right)$is a 2D Gaussian kernel: $G\left( x,y,\sigma\right)= \frac{1}{2\pi\sigma^{2}}e^{\frac{-(x^{2}+y^{2})}{2\sigma^{2}}}$. In this formula, σ represents the expected vessel diameter. Optimization of the Frangi filter for each image type based on the expected diameter of vessels is an important step since it can impact the measured diameter. Similarly to other works [41,60], we have used a multi-scale approach which allows us to enhance a range of vessel diameters by iterating over a range of σ values from 0 to σ_max_, where σ_max_ is the expected maximum vessel diameter. Selection of too large a value for σ_max_ will lead to artificial vessel dilation whereas underestimation will lead to insufficient SNR enhancement. While the range of σ values can be estimated based on *a priori* knowledge of the expected range of vessel diameters within an image and verified empirically, we determined that measuring the apparent full width at half maximum (FWHM) of the vessel diameter directly from the OCT image provided a more quantitative benchmark for optimization. For example, [60] used values in the range 1-10 for σ corresponding to vessel diameters in the range 10-100 µm in skin in the crook of the elbow and knee. Through our analysis, we determined that σ values in the range 1-8, corresponding to vessel diameters in the range 10-80 µm, is more appropriate for our measurement location on the hand. The results of our analysis are shown in S1 Fig**.** The thick black line indicates the intensity profile measured directly from the OCTA MIP image. For all values of σ_max_, the background signal level outside of the vessel has been reduced, but for σ_max_ ≤ 4, the signal intensity of the vessel itself is also reduced. For larger values of σ_max_ ≥ 12, the apparent vessel diameter is overestimated. By inspection, we chose the median value of σ_max_= 8 as representing the best trade-off between accurate vessel diameter and vessel enhancement for the range of vessel diameters present in our images.
