# Supplementary figures and images for: OCTAVA: An open-source toolbox for quantitative analysis of optical coherence tomography angiography images
Source: PLoS One. 2021 Dec 9;16(12):e0261052. doi: 10.1371/journal.pone.0261052 (PMC8659314; doi:10.1371/journal.pone.0261052)

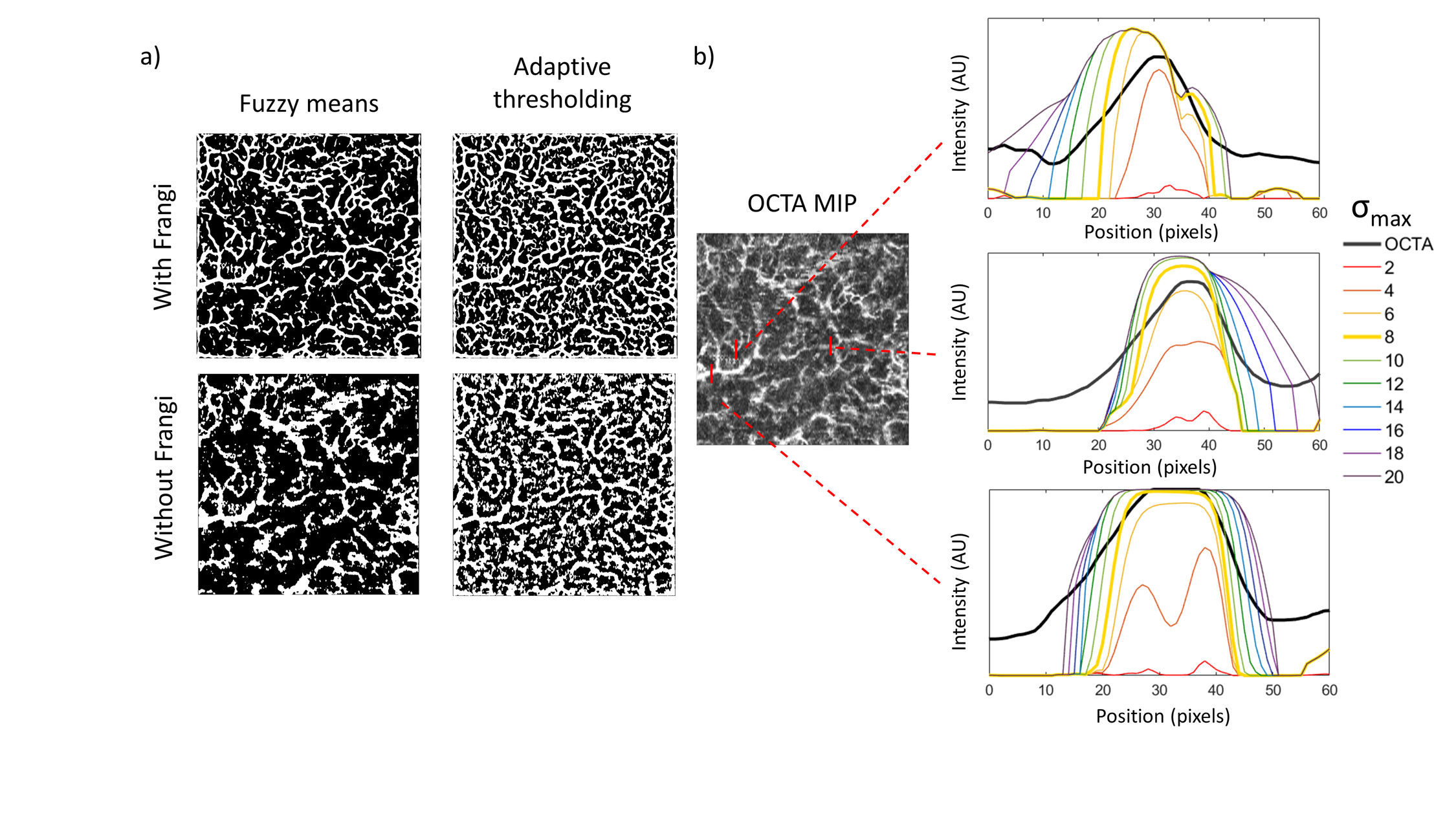

Supplement: S1 Fig — a) Indication of the impact of the Frangi filter on the interconnectivity of the vascular network after segmentation using two methods, as labeled: in both cases, σmax = 8. b) Demonstration of the impact of the Frangi filter on SNR and apparent vessel diameter for a range of σmax values. Red lines on the OCTA MIP image (center) mark three vessels with different diameters and OCTA and filtered line profiles in the graphs on the right as examples of the impact of the Frangi filter on apparent diameter in different scenarios. The OCTA MIP image dimensions are 5 mm × 5 mm. (TIF) [file pone.0261052.s002.tif]
